# Prioritizing children’s mental health amidst Sudan’s humanitarian crisis: policy recommendations for immediate action

**DOI:** 10.1186/s13034-023-00640-9

**Published:** 2023-08-10

**Authors:** Sarah Hashim Mohammed Osman, Abdulqadir J. Nashwan

**Affiliations:** 1 Faculty of Medicine, University of Khartoum, Khartoum, Sudan; 2 Nursing Department, Hamad Medical Corporation, P.O. Box 3050, Doha, Qatar

**Keywords:** Sudan conflict, Child health, Humanitarian crisis, Policy intervention, Health service accessibility

## Abstract

The humanitarian crisis precipitated by the ongoing conflict in Sudan poses profound risks to the health and welfare of the country’s children. This paper explores essential policy interventions to safeguard child mental health services under these challenging circumstances. Crucial strategies include enhancing healthcare accessibility for children and their caregivers, promoting education, and improving household living conditions. Additionally, it is vital to provide improved access to information about nutritious food and strengthen health systems in areas directly exposed to conflict. Cooperation with international aid organizations is paramount to delivering medical supplies to functioning health facilities. The paper also recommends partnerships with local non-governmental and humanitarian organizations to execute public health programs effectively. These multi-faceted policy measures underscore the importance of a comprehensive response to ensure the health and well-being of children amid the turmoil in Sudan. Through these strategies, we aim to provide a blueprint for policymakers and humanitarian organizations to mitigate the devastating impacts of the conflict on the country’s most vulnerable population.

The ongoing conflict in Sudan is wreaking havoc on the country’s health services, with profound effects that extend far beyond the immediate violence. In Khartoum alone, the death toll has reached 700, with 6,000 others suffering injuries [1, 2]. Moreover, the instability has provoked a mass displacement crisis; 248,300 individuals have sought refuge in neighboring countries, while an additional 843,000 are internally displaced within Sudan [3].

The humanitarian need in the country is staggering, with an estimated 24.7 million individuals requiring assistance [1]. Of the 820 hospitals spread across the country’s 18 states, 134 reside in Khartoum state and have sustained severe damage [4, 5]. In addition, the political turmoil and continuous conflict in Khartoum have exacerbated the health system’s challenges, leading to budget cuts, medical activity disruption, shortages of skilled health workers and supplies, and widespread destruction of specialized health facilities [1].

For those displaced, their plight is characterized by poor living conditions, terror, property loss, and the burden of seeking care in distant, resource-poor areas [6]. Despite their efforts, international aid organizations are facing immense challenges in aiding the conflict-affected population [6].

According to the United Nations International Children’s Emergency Fund (UNICEF), the escalating conflict in Sudan is exacerbating an already dire humanitarian situation for children [7]. At least nine children have been killed, and seven others injured due to heavy weaponry and explosive devices in civilian areas [7]. With approximately 1.4 million children displaced, their basic needs of food, healthcare, water, and education are jeopardized [7]. Armed forces are attacking and occupying schools, healthcare facilities, and water infrastructures [7]. Children, particularly girls, are increasingly at risk of exploitation, abuse, and sexual violence [7]. However, UNICEF’s programs, which focus on health, nutrition, water, education, and child protection, are underfunded, limiting its ability to respond to the urgent needs of children in Sudan fully.

The conflict has notably impacted pediatric health services, creating numerous barriers to children’s nutritional and immunization services accessibility. The World Health Organization (WHO) data indicates that conflict-affected countries frequently grapple with low immunization coverage and outbreaks of vaccine-preventable diseases (VPDs) [6]. In addition, many specialized hospitals and clinics have been demolished, leading to overcrowding and understaffing at the remaining facilities [8]. Adding to this, the disruption of public health programs due to movement restrictions, tense security situations, and targeting of health workers has hindered mass vaccination campaigns and disrupted health services [9]. The shortage of nutritional supplies has also resulted in malnutrition, growth defects, immune and metabolic defects, malaria, diarrheal disease, measles, and lower respiratory infections epidemics [9, 10]. These circumstances have led to a significant decline in children’s quality of care.

Moreover, children are exposed to violence, starvation, illness, and loss of parents, leading to abandonment, abduction, displacement, forced soldiering, and poverty [11, 12]. Physical trauma may result in permanent disability, while mental and behavioral health sequelae, such as depression and post-traumatic stress disorder (PTSD), are prevalent [12, 13], particularly given the lack of mental health services outside of Khartoum state. The dire maternal health situation compounds the problem, with premature babies at risk of developing intellectual disabilities, cerebral palsy, and hearing and visual impairments [14]. These potential risks pose long-term threats to their physical and mental health.

The escalating conflict in Sudan presents a pressing humanitarian crisis, with the most profound implications falling upon the country’s most vulnerable group: children. It is a moral imperative to respond as a society, calling for decisive policy interventions that prioritize and safeguard maternal and child health services amidst this chaos. Access to healthcare services for children and their caregivers needs immediate improvement. In a displacement and widespread insecurity setting, ensuring that medical facilities are reachable and equipped to handle pediatric and maternal needs is paramount. This multi-faceted strategy could include mobile clinics, telehealth services, and adequately trained community health workers. Education, particularly for girls, must also be upheld. In conflict settings, schools offer knowledge and provide a semblance of routine and stability for children. They can be hubs for distributing nutritional meals and implementing health programs, including vaccinations and psychological support. Improvements in household living conditions are also crucial. Displaced and conflict-affected families often live-in unsanitary conditions, increasing the risk of diseases. Policies that facilitate the provision of clean water, sanitation facilities, and safe shelters are necessary to mitigate these risks. Access to accurate information regarding nutritious food is another area of focus. Public health campaigns that educate caregivers on the importance of balanced diets and breastfeeding can help prevent malnutrition, which is especially critical in conflict-impacted regions. Policymakers also have a responsibility to fortify the health systems in conflict-exposed areas. This can involve infrastructural investments, training local healthcare professionals, and ensuring essential medications and medical equipment are continuously available. Collaborating with international aid organizations can enhance this effort. By facilitating their entry and operations, the government can quickly deliver much-needed medical supplies to operational health facilities. Finally, partnering with local non-governmental and humanitarian organizations can prove beneficial. These groups have invaluable ground-level insights and connections, enabling more effective implementation of public health programs. Moreover, they can serve as advocates for the local communities, voicing their needs and concerns to a larger audience.

In conclusion, the Sudan conflict is not just a political issue—it is a human issue that demands immediate, comprehensive responses to safeguard the health and well-being of its most vulnerable populations, especially children.

## Data Availability

Not applicable.
